# Japanese encephalitis accompanied by cerebral venous sinus thrombosis: a case report

**DOI:** 10.1186/1471-2377-12-43

**Published:** 2012-06-19

**Authors:** Min Jia, Nian Xiong, Jinsha Huang, Youpei Wang, Xiaowei Zhang, Zhentao Zhang, Xuebing Cao, Zhicheng Lin, Tao Wang

**Affiliations:** 1 Department of Neurology, Union Hospital, Tongji Medical College, Huazhong University of Science and Technology, 1277 Jiefang Road, Wuhan, 430022, Hubei, China; 2 Department of Neurology, The Central Hospital in En Shi, 158 Wu Yang Ave, 445000, En Shi, Hubei, China; 3 Department of Neurology, Renmin Hospital of Wuhan University, Wuhan, 430060, China; 4 Department of Psychiatry, Harvard Medical School; Division of Alcohol and Drug Abuse, and Mailman Neuroscience Research Center, McLean Hospital, Belmont, MA, 02478, USA; 5 Harvard NeuroDiscovery Center, Boston, MA, 02114, USA

**Keywords:** Cerebral venous sinus thrombosis, Epidemic encephalitis type B, Magnetic resonance venography, Encephalitis type B-specific IgM antibody, Case report

## Abstract

****Background**:**

Cerebral venous sinus thrombosis (CVST) is a relatively rare cerebrovascular condition which accounts for 0.5% of all strokes. Risk of CVST has been documented in patients with numerous conditions including central nervous system infections, however, Japanese encephalitis (JE, epidemic encephalitis type B) with CVST has not been reported previously.

****Case Presentation**:**

Here, we present a case of JE with CVST in a 17-year-old man. On admission, the patient was initially diagnosed as intracranial infection, and soon after, brain magnetic resonance (MR) imaging (MRI) and MR Venography (MRV) confirmed the diagnosis of CVST. Moreover, the blood JE-specific IgM antibody which proved weakly positive at first, turned positive one week later. Consequently, our patient was diagnosed as CVST accompanied by JE. Anticoagulant and anti-infective therapy were initiated, which eventually lead to gradual recovery of the patient.

****Conclusions**:**

To our knowledge, this is the first case report of CVST associated with JE. MRI and MRV represent a prime method for the diagnosis of CVST, while the positivity of JE virus IgM antibody, especially increased antibody levels within a short period, is of great significance to diagnose JE. The early diagnosis and timely treatment of this potentially lethal condition would improve its prognosis significantly.

## Background

Japanese encephalitis (JE, also known as Epidemic encephalitis type B) is an acute central nervous system disease caused by mosquito-borne Japanese encephalitis virus infection. It is a seasonal epidemic disease and predominantly affects human in July, August, and September each year. The typical clinical features of JE are of acute onset, accompanied by high fever, headache, impaired consciousness, convulsions and meningeal irritation [1].

Cerebral venous sinus thrombosis (CVST) is a rare cerebrovascular disorder which accounts for 0.5% of all strokes [2]. CVST is commonly misdiagnosed or delayed-diagnosed, as its various modes of onset are atypical and is characterized by nonspecific clinical features such as headache, seizures, altered consciousness, papilloedema and any of the other symptoms of stroke [2]. The prothrombotic risk factors include, infective conditions (e.g. central nervous system infections, mastoiditis, vasculitis, and ear, sinus or face infection as well as other systemic inflammatory disorders) and non-infective conditions (e.g. pregnancy and puerperium, haematological diseases, oral contraceptives, head trauma, dehydration and systemic lupus erythematosus) [2,3]. There are many viral infections documented to be associated with stroke, such as varicella-zoster virus [2,3]. Furthermore, cerebral venous thrombosis (CVT) was also identified in a patient presenting with fever convulsions, and associated herpes simplex encephalitis [2,3]. Viral hepatitis is being regarded as a rare infective cause of CVT [2,3]. JE virus is also a common cause for cerebral ischemia in children [2,3]. However, JE with CVST has not yet been reported literally.

Presently, a case of JE with CVST, has been diagnosed in the Department of Neurology, Union Hospital, Tongji Medical College, Huazhong University of Science and Technology (TMC&HUST), Hubei, China, and we hereby describe the case in detail.

## Case presentation

A 17-year-old man, soldier, was admitted at the Department of Neurology, Union Hospital, TMC&HUST with a history of severe headache and high fever for 4 days. The symptoms started abruptly while he was undergoing military training. His body temperature was 39.8°C, without any other symptoms. The symptoms were controlled by administration of routine symptomatic treatments. However, the headache symptom recurred 3 days later, along with nausea and vomitting. His cerebral computed tomographic (CT) scan was normal. During his admission to our department on August 4^th^, 2010, his symptoms developed progressively, with altered consciousness, dysphoria and urinary incontinence.

The patient appeared to be delirious and confused during the interview. His body temperature, pulse, respiration and blood pressure were 39.2°C, 105 beats per minute, 36 breaths per minute, and 128/70 mmHg (1 mm Hg = 0.133 kPa), respectively. The physical examination did not indicate any abnormalities in chest or abdomen. The neurological examination revealed that his pupils were equal in size (diameter = 2 mm), round in shape, and the pupillary light reflex was diminished on both sides. Conjunctival edema and limb-associated spontaneous activity was observed. Tendon reflexes of the limbs were symmetrically weakened, while the right Babinski sign was positive. Neck stiffness was found (the chin, 3 horizontal finger apart from the chest). The remainder of the examination could not be completed as the patient was uncooperative.

**Figure 1:**
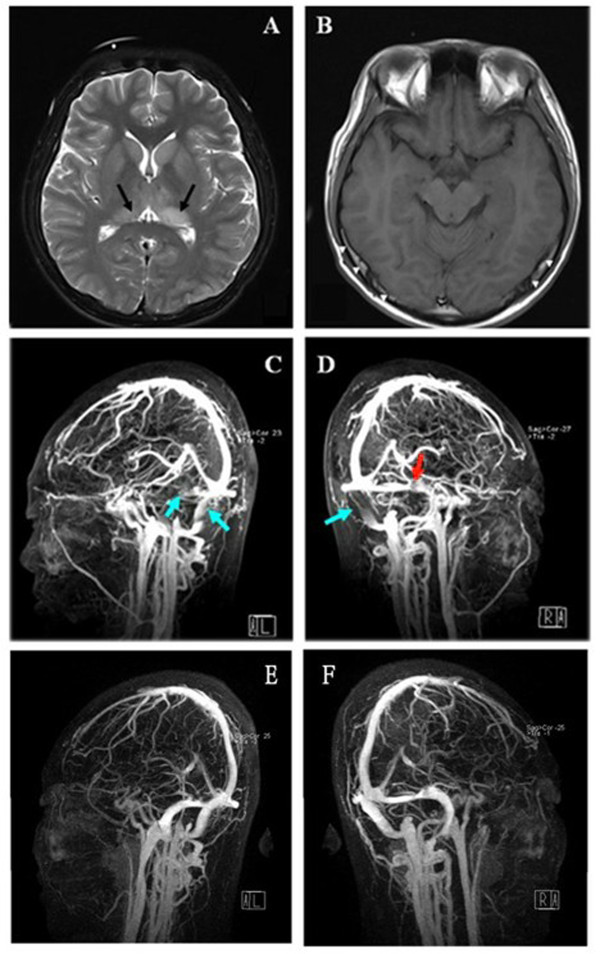
**MR imaging of the patient before and after treatment.** (**A**) Axial T2-weighted images showed the swelling of bilateral thalamus. (**B**) Contrast Enhanced MRI scan revealed sigmoid sinus and transverse sinus filling defect (white arrows), indicating venous thrombosis. (**C** and **D**) The MRV scan indicated absence of signal in the bilateral sigmoid sinuses and left transverse sinuses before treatment (blue arrows). (**D**) Red arrow showed an arachnoid granulation not venous thrombosis in the right transverse sinus. (**E**, **F**) The repeat MRV scan demonstrated only a few low signal areas in the left sigmoid sinus after treatment.

The electrocardiogram showed sinus rhythm with normal PR and QTc intervals. The lung CT scan revealed small irregular patchy shadows at inferior pulmonary lobe both sides. B-mode ultrasound scan of the abdomen was normal. Urinalysis showed 1+ albumin. Widal test, Plasmodium test, blood and urine cultures were negative. Blood cell count indicated that white-cell count was 12.27 x 10^9^/L (reference range, 4–10 x 10^9^/L), neutrophil count was 10.31 x 10^9^/L (reference range, 2–7 x 10^9^/L), platelets and hemoglobin were normal. Other laboratory tests, including erythrocyte sedimentation rate, prothrombin time and activated partial thromboplastin time, yielded normal results. We failed to perform a Lumbar Puncture to analyze his cerebrospinal fluid (CSF) on admission, as the patient was uncooperative. Nevertheless, brain MRI displayed the gyral swelling of bilateral occipital and temporal lobes as well as thalamus, while the contrast Enhanced MRI scan revealed remarkable enhancement of leptomeninges. In addition, sigmoid sinus and transverse sinus filling defect were observed, indicating venous thrombosis. Likewise, MRV scan further demonstrated thrombosis in the bilateral sigmoid sinuses and left transverse sinuses, confirming the diagnosis of CVST (Figure 1 A, B, C, D). The blood JE-specific IgM antibody (InBios’ JE Detect™ IgM ELISA kit, InBios International, Inc., Seattle, Washington, USA) was weakly positive, and the repeated test showed positive results one week later. We performed a Lumbar Puncture on August 18, 2010 as the psychiatric symptoms of our patient then came under control. The CSF tests showed: pressure 80 mmH_2_O, white blood cell (WBC) count 7 x 10^6^/L, protein 0.57 g/L, glucose 3.73 mmol/L, and chloride 118.8 mmol/L. Consequently, our patient was diagnosed as CVST as well as JE.

Low-molecular-weight heparin and then warfarin was administered to the patient. Other symptomatic therapeutic measures (including dehydration, antipsychotic therapy, antiinflammatory, antivirus, and body temperature control), and supportive therapeutic measures were taken simultaneously. As a result, the patient’s headache was relieved, chemosis disappeared, and the body temperature gradually dropped to normal level without the appearance of any mucocutaneous petechiae or purpura. The psychiatric symptoms were under control as well. The patient was discharged on August 23^th^, 2010 and transferred to a local hospital for further treatment. The repeat brain MRI scan revealed mild oedema over bilateral thalamus. Compared to the MRI result of August 6^th^, 2010, the lesion diminished significantly. The MRV demonstrated only a few low signal areas in the left sigmoid sinus, without detecting any filling defect in left transverse sinus (Figure 1 E, F).

## Discussion and conclusions

The incidence of CVST, a rare cause of stroke, is approximately 2–4 cases per million population per year [2,4]. The clinical manifestations of CVST are nonspecific, highly variable, similar to the clinical presentations of several other clinical conditions, such as arterial stroke, meningeal or cerebral inflammatory disease, brain abscess, cerebral vasculitis and so on [4]. In the past, the diagnosis of CVST highly depended on digital subtraction angiography (DSA), which is the ‘gold standard’ and commonly used for endovascular treatment. However, DSA is an invasive and expensive approach. Compared to DSA, MRI and MRV grant the advantages of noninvasiveness, no radiation, convenience, and especially easy to follow up. Thus, the combination of MR features including thrombosed vessel from MRI and nonvisualization of the same vessel from MRV have been considered to be the best diagnostic information for CVST. The patient had been initially diagnosed as intracranial infection and viral encephalitis, and afterwards MRI provided an important clue to revise the diagnosis quickly, thus further confirming the advantage of MRI and MRV in the diagnosis of CVST.

In addition to intracranial hypertension, our patient’s other characteristics can be summarized as follows: 1) A 17 years old man, acute onset of symptoms; 2) High fever (39°C), and confusion; 3) Abnormal reflexes and meningeal irritation positive; 4) Summer season, with a history of mosquito bites; 5) MR imaging of brain showed bilateral thalamic swelling, enhanced scan showed marked leptomeningeal enhancement; 6) White-cell counts and neutrophil counts raised. According to its characteristics, we further investigated his serologic JE-specific IgM antibodies twice, the first was weakly positive, and the second result showed positivity one week later, suggesting that the antibody levels increased.

Due to its complexity, JE virus isolation is not a routine method for laboratory diagnosis. Detecting serologic JE virus antibodies was currently the main laboratory diagnostic method. As the patient was a soldier, we were unable to obtain epidemiological data on admission. Later, one of his comrades (living in different camps in the same county) who appeared to have similar symptoms, was also diagnosed with epidemic encephalitis type B, thus conferring evidence to the location of mosquito borne disease in their case. We further performed a Lumbar Puncture when the psychiatric symptoms of our patient were under control. As his serologic JE-specific IgM antibodies were positive, we did not further investigate his JE-specific IgM antibodies in CSF. This case reminds us that the possibility of JE should be considered if a patient is with high fever, headache, and impaired consciousness in late summer and autumn. For the suspicious cases, the epidemiological data should be repeatedly obtained and investigated as well as reported to CDC immediately. Unlike other viral infections, most of JE patients, had peripheral white blood cell count and neutrophil count increased. Research has shown that neuroimaging was helpful in making early diagnosis of JE by showing characteristic involvement of bilateral thalami [5]. On the other hand, imaging of CVST can also show abnormal signal in unilateral or bilateral thalamus. Therefore, it was hard to determine whether JE or CVST, or both acted as probable causes of the MR imaging demonstrating bilateral thalamic signal abnormality.

Lateral sinus thrombosis (LST) is often associated with mastoiditis and otitis media, or infection that spreads to sigmoid sinus through a dehiscence in the overlying bone, or the direct dissemination of the infection through the neighboring eroded bone [2,4,6]. Our patient had no history of mastoiditis or otitis media, his white-cell counts and neutrophil counts were elevated while platelet level, prothrombin time and activated partial thromboplastin time were normal. Moreover, the antiphospholipid antibodies test was negative. Unfortunately, further assessment of procoagulant state including protein C, protein S, antithrombin III, factor V, were not performed. So, we speculated that combination of systemic infection and dehydration might contribute to the hypercoagulability of this patient. In addition, the possible mechanisms underlying CVT in JE viral infection can be summarized as follows: 1) the penetration of virus through the endothelial walls contributed to an increase in the permeability of the cerebral vessels (capillaries and precapillaries)[2,4,6]; 2) JE virus could induce increases in the level of interleukin-6 and tumor necrosis factor-α which are risk factors for thrombosis [2,4,6].

In summary, the clinical manifestations of CVST are nonspecific, making this disorder prone to misdiagnosis. MRI and MRV represent a prime method for the diagnosis of this disease. Even though the initial presentation of the disease could be severe, partial or complete recovery is possible, depending on early recognition. The positivity of JE virus IgM antibody, and particularly, increased antibody levels within a short period, is of great significance to diagnose JE. Hence, JE-specific IgM antibody test on the suspicious cases which lack reliable epidemiological data and those which are difficult to diagnose clinically, is necessary. Due to widespread application of the JE vaccine, the number of JE cases has significantly decreased worldwide. It is necessary for us to study JE again and to continue educating the public about this serious infected disorder. To our knowledge, this is the first report of JE with CVST. The early diagnosis and timely treatment of this potentially lethal condition would benefit the patients and the whole society.

## Consent

Written informed consent was obtained from the patient and his parents for publication of this Case report and any accompanying images. A copy of the written consent is available for review by the Series Editor of this journal.

## Abbreviations

CVST, Cerebral venous sinus thrombosis; JE, Japanese Encephalitis, encephalitis type B; MRI, MR imaging; MRV, MR venography; CT, computed tomography; CSF, cerebrospinal fluid; DSA, digital subtraction angiography; LMWH, low molecular weight heparin; TJMC & HUST, Tongji Medical College, Huazhong University of Science.

## Competing interests

We confirm that we have read the Journal’s position on issues involved in ethical publication and affirm that this report is consistent with those guidelines. None of the authors have any conflict of interest to disclose.

## Authors’ contributions

MJ, NX, JH, YW, XZ, ZTZ, XC, ZCL, TW contributed to the conception and design. MJ, NX, JH, XZ, ZTZ took care of collecting the clinical information. MJ, NX, ZTZ, TW analyzed and interoperated the MR images. MJ, NX, JH, WT, ZCL coordinated and helped to draft the manuscript. All authors have read, revised and approved the final manuscript.

## Pre-publication history

The pre-publication history for this paper can be accessed here:

http://www.biomedcentral.com/1471-2377/12/43/prepub

## References

[B1] Wang H Li Y Liang X Liang G Japanese encephalitis in mainland china Jpn J Infect Dis 2009 62 5 331 336 19762980

[B2] Bousser MG Ferro JM Cerebral venous thrombosis: an update Lancet Neurol 2007 6 2 162 170 10.1016/S1474-4422(07)70029-7 17239803

[B3] Kashyap AS Anand KP Kashyap S Thrombosis of the cerebral veins and sinuses N Engl J Med 2005 353 3 314 315 16038059

[B4] Stam J Thrombosis of the cerebral veins and sinuses N Engl J Med 2005 352 17 1791 1798 10.1056/NEJMra042354 15858188

[B5] Prakash M Kumar S Gupta RK Diffusion-weighted MR imaging in Japanese encephalitis J Comput Assist Tomogr 2004 28 6 756 761 10.1097/00004728-200411000-00005 15538147

[B6] Bousser MG Cerebral venous thrombosis: diagnosis and management J Neurol 2000 247 4 252 258 10.1007/s004150050579 10836615

